# Fabrication of aluminum alloy functionally graded material using directional solidification under an axial static magnetic field

**DOI:** 10.1038/s41598-018-26297-5

**Published:** 2018-05-21

**Authors:** Shaodong Hu, Annie Gagnoud, Yves Fautrelle, Rene Moreau, Xi Li

**Affiliations:** 1 State Key Laboratory Advanced Special Steel, Shanghai University, Shanghai, 200072 People’s Republic of China; 2 SIMAP-EPM-Madylam/G-INP/CNRS, PHELMA, 38402 St Martin d’Heres Cedex, France

## Abstract

Aluminum alloy *in situ* functionally graded materials (FGMs) have been successfully fabricated using directional solidification under an axial static magnetic field. Al-Zn, Al-Ni and Al-Cu alloys with a hypereutectic composition were selected to produce FGMs. Experimental results show that the graded composition of the primary phases (i.e., Zn, Al_3_Ni and Al_2_Cu) is obvious along the longitudinal section of the sample. The graded composition of the primary phases could be controlled by the value of the magnetic field, growth rate and temperature gradient. A proposed model and simulations are carried out to explain the origin of the graded composition of the primary phases in FGMs during directional solidification under an axial static magnetic field. It should be attributed to the combined actions of heavier species migration under gravity force and thermoelectric (TE) magnetic convection under magnetic field. Furthermore, it can be found that the magnetic field can induce the columnar FGMs to change into equiaxed FGMs. This work not only presents a new approach to fabricate FGMs using the directional solidification under an axial static magnetic field but also deeply understands the effect of the solute migration and temperature distribution on the crystal growth during directional solidification.

## Introduction

Functionally graded material (FGM) is a composite of multi-phase microstructure. FGMs have attracted long-term attentions since Japan’s FGMs programmes began. The microstructures and properties of FGMs vary with locations, which can meet the special engineering applications, such as in aerospace or nuclear power generation require parts^1–5^. Many physical and chemical methods have been used to make FGMs^6–11^, such as vapor deposition, ultrasonic welding, fusion welding, layer/disk re-melting, powder metallurgy and centrifugal methods.

Magnetic field, as an external field method, has also been used to fabricate FGMs. The application of gradient high magnetic field successfully fabricated FGMs^12–15^. However, so far, few studies have used static magnetic fields to fabricate FGMs. In the past long time, the static magnetic field was considered to suppress or damp the liquid motion due to the braking effect of the Lorentz force^16,17^. Nevertheless, a large number of studies have confirmed that the static magnetic field can cause thermoelectric (TE) magnetic convection in recent years^18–22^. The static magnetic field, causing TE magnetic convection and solute migration during directional solidification, can be applied to fabricate FGMs during directional solidification.

The present work describes an *in situ* technique to fabricate Al alloy FGMs (*i*.*e*., Al-Zn, Al-Ni and Al-Cu alloys) using directional solidification under an axial static magnetic field. The morphology and microstructure of the Al alloy FGMs were observed with an optical microscope. A proposed model and simulations are carried out to explain the origin of the graded composition of the primary phases in FGMs. Furthermore, the magnetic field can induce columnar FGMs to change into equiaxed FGMs. This work not only presents a new approach to fabricate FGMs using the directional solidification under an axial static magnetic field but also deeply understands the effect of the solute migration and temperature distribution on the crystal growth during directional solidification.

## Experimental Procedure

The original samples with an 80 mm diameter of Al-96 wt.%Zn, Al-12 wt.%Ni and Al-40 wt.%Cu alloys were prepared by pure Al (99.99 wt.%), Zn (99.99 wt.%), Ni (99.99 wt.%) and Cu (99.99 wt.%) in a vacuum induction furnace. The cast samples were cut by electro-discharge machining and enveloped in the high-purity corundum crucibles for the directional solidification experiment. The inner diameter and length of the corundum crucible are 3 mm and 100 mm, respectively. The length of the sample is 80 mm. The experimental apparatus consists of an electromagnet, a Bridgman-Stockbarger type furnace, a temperature controller and a pulling velocity control unit. A schematic illustration of the experimental apparatus can be found in our previous study^23^. The superconductor magnet can produce an axial static magnetic field and the magnetic field intensity is up to 5 T. The heating temperature in the furnace can reach 1773 K. The liquid Ga-In-Sn metal (LMC) with water cooled cylinder was used to cool down the sample. The samples were directionally solidified in the furnace at various pulling velocities. The pulling speed can be adjusted from 0.5 μm/s to 10000 μm/s. During directional solidification, the initial solidification interface of the sample was always put into the center of the superconductor magnet. The longitudinal and transverse sections of directionally solidified samples were cut by electro-discharge machining. The samples were grinded, mechanically polished and chemically etched with 1.5% HCl, 2.5% HNO_3_, 1% HF and 95% (vol.%) H_2_O solution. The structure was observed with a Leica DM 6000 M optical microscope. In order to clearly display the primary phase of the whole directionally solidified sample, the brightness, sharpness and contrast of the photographs were adjusted, so that eutectic structure may become black. The unevenly distributed eutectic structure can make it difficult to distinguish eutectic and primary phase after the adjustment. Therefore, the eutectic structure at the upper part of the sample was cut. The distance from the initial solidification interface to the end of the solidified sample is taken as the total solidification distance. The ratio of the distance solidified to the total length of initial melt column is taken as the fraction distance solidified.

## Experimental Results

**Figure 1:**
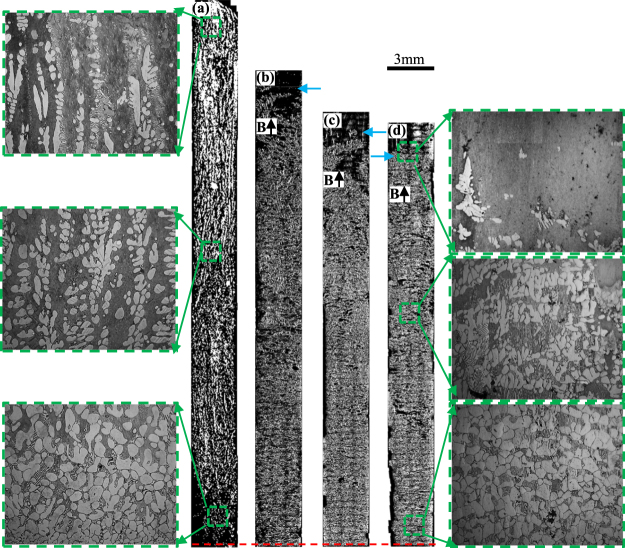
The longitudinal microstructure of Zn/Zn-Al gradients under various magnetic fields at a 7 μm/s growth rate during directional solidification. (**a**) 0 T; (**b**) 1 T; (**c**) 3 T; (**d**) 5 T. Blue arrows marking the interface of the primary phases and red dotted line showing the initial solidification interface.

**Figure 2:**
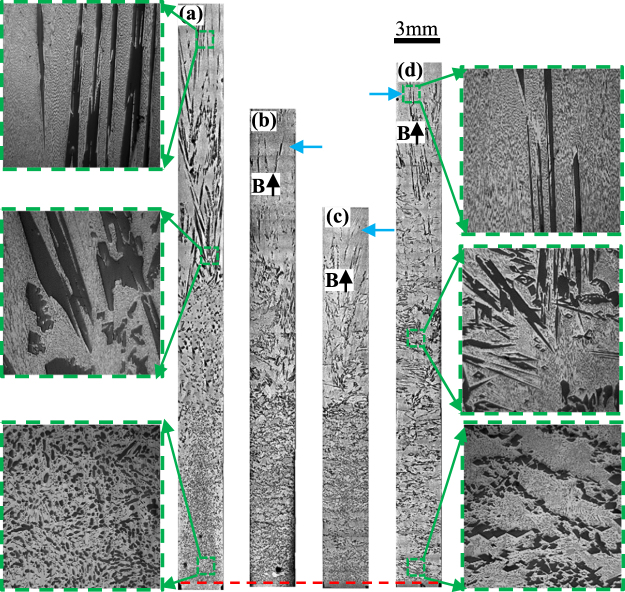
The longitudinal microstructure of Al_3_Ni/Al-Al_3_Ni gradients under various magnetic fields at a 10 μm/s growth rate during directional solidification. (**a**) 0 T; (**b**) 0.5 T; (**c**) 1 T; (**d**) 3 T. Blue arrows marking the interface of the primary phases and red dotted line showing the initial solidification interface.

**Figure 3:**
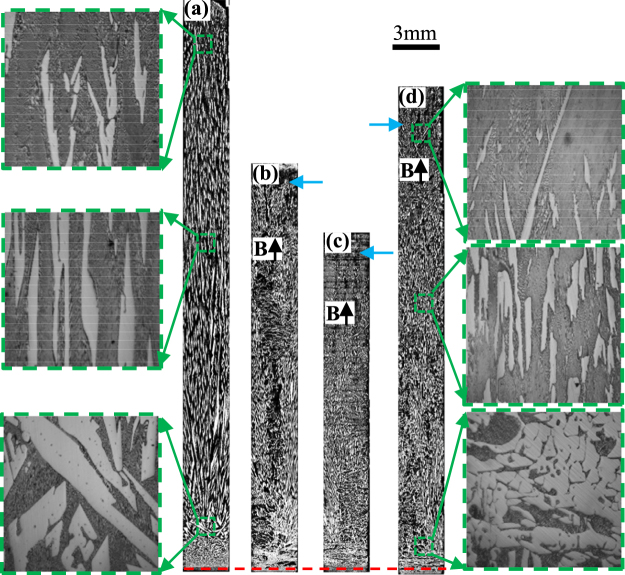
The longitudinal microstructure of Al_2_Cu/Al-Al_2_Cu gradients under various magnetic fields at a 10 μm/s growth rate during directional solidification. (**a**) 0 T; (**b**) 0.5 T; (**c**) 1 T; (**d**) 3 T. Blue arrows marking the interface of the primary phases and red dotted line showing the initial solidification interface.

**Figure 4:**
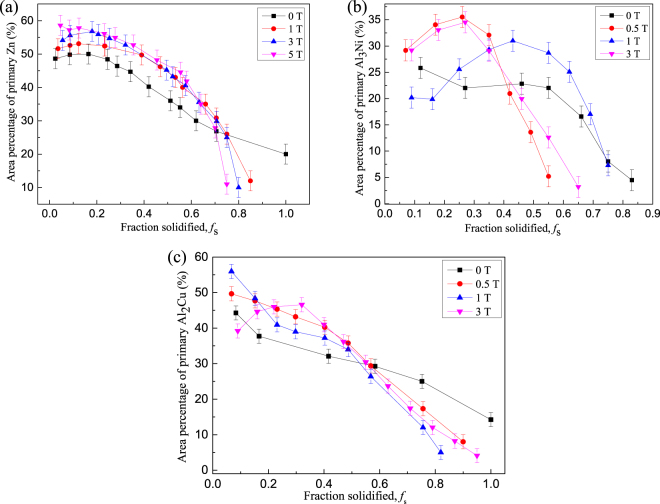
The variation in area percentage of the primary phases as a function of fraction distance solidified under various magnetic fields in the directionally solidified Al alloys. (**a**) Al-96 wt.%Zn; (**b**) Al-12 wt.%Ni; (**c**) Al-40 wt.%Cu.

Figure 1 shows the longitudinal microstructures of directionally solidified hypereutectic Al-96 wt.%Zn alloys at a growth rate of 7 μm/s and a temperature gradient of 90 K/cm under the magnetic fields of 0 T, 1 T, 3 T, 5 T, respectively. The solidified microstructures consist of primary phase Zn (white) and lamellar Al-Zn eutectic phase. The primary phase is distributed in the matrix of the lamellar Al-Zn eutectic phase. One can notice that there is no obvious change of the distribution of the primary Zn along the longitudinal section of the sample in the absence of the magnetic field. However, the applied magnetic field results in the modification of the primary Zn phase distribution. The enrichment of the primary Zn phase occurs at the bottom of the sample. When the growth length increases to certain extent, at the top of the sample, the primary Zn phase disappears and the eutectic microstructure will form. Thus, the graded composition of the primary Zn phases along the longitudinal section of the sample is generated. Furthermore, it can be found that the graded composition of the primary Zn phases is enhanced by the magnetic field. Figures 2 and 3 show the longitudinal microstructures of directionally solidified hypereutectic Al-12 wt.%Zn and Al-40 wt.%Cu alloys at a growth rate of 10 μm/s and a temperature gradient of 90 K/cm under the various magnetic fields, in the value of 0 T, 0.5 T, 1 T and 3 T. The same phenomenon can also be found that the applied magnetic field leads to the increase of the enrichment degree of the primary phases (black primary Al_3_Ni and white primary Al_2_Cu) at the bottom of the samples. Meanwhile, the eutectic microstructures form at the top of the samples where the primary phases disappear. However, the enrichment of the primary phases is not always enhanced by the magnetic field for both alloys. When the magnetic field is more than 1 T, the enrichment of the primary phases at the bottom of the samples is weakened by the magnetic field. The relationship between the area percentage of the primary phase and fraction distance solidified in Figs 1–3 is shown in Fig. 4. It is noted that the area percentage of the primary phase tends to decrease as the fraction distance solidified increases for all the alloys in the absence of magnetic field. When the magnetic field is applied, the downward trend of the primary phase area percentage with the fraction distance solidified is increased by the magnetic field for directionally solidified hypereutectic Al-96 wt.%Zn alloy. However, for the other two alloys, the downward trend is increased first and then is decreased. Thus, the microstructures of the Al alloys vary with locations, which shows the evident graded composition profile along the longitudinal sections of the samples during directional solidification. In this way, *in situ* FGMs can be successfully fabricated by the axial static magnetic field in the Al hypoeutectic alloys.

**Figure 5:**
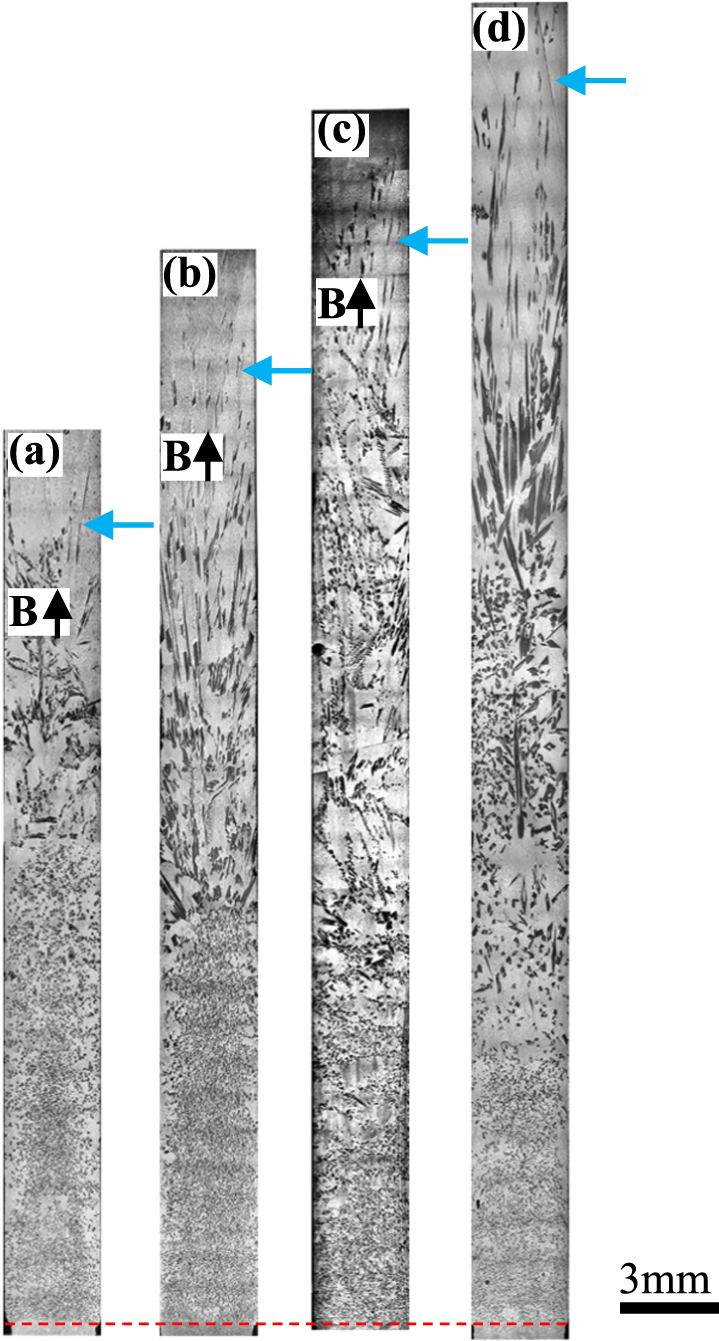
The effect of the growth rate on the longitudinal microstructure of Al_3_Ni/Al-Al_3_Ni gradients in directionally solidified Al-12 wt.%Ni. (**a**) 0.1 T, 5 μm/s; (**b**) 0.1 T, 10 μm/s; (**c**) 0.1 T, 15 μm/s; (**d**) 0 T, 15 μm/s. Blue arrows marking the interface of the primary phases and red dotted line showing the initial solidification interface.

**Figure 6:**
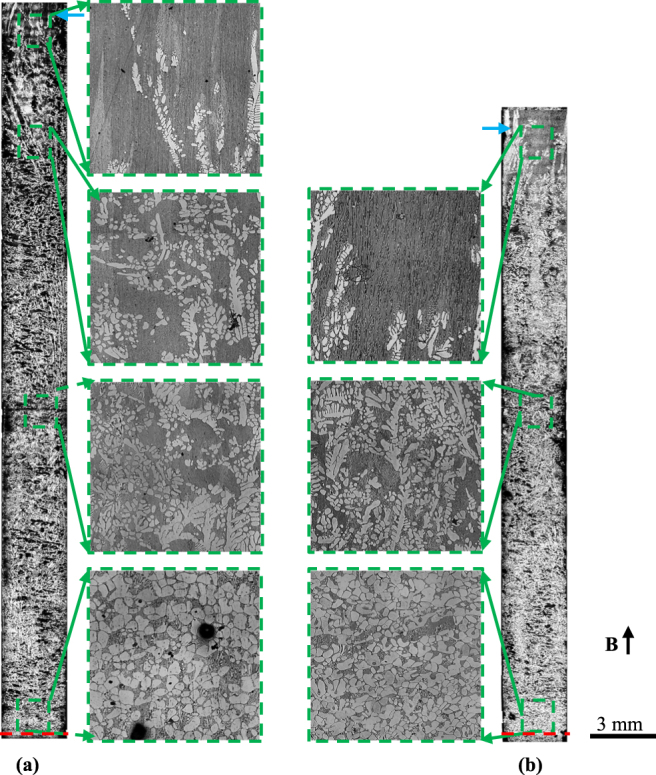
The effect of the temperature gradient on the longitudinal microstructure of Zn/Al-Zn gradients at a 3 μm/s growth rate in directionally solidified Al-96 wt.%Zn under 0.5 T magnetic field. (**a**) 60 K/cm; (**b**) 120 K/cm. Blue arrows marking the interface of the primary phases and red dotted lines showing the initial solidification interface.

Moreover, the influences of other solidification parameters, *i*.*e*., the growth rate and temperature gradient, on the distribution of the primary phase during directional solidification under the magnetic field are studied. The results show that the graded composition profile in FGMs can be changed by the solidification parameters. Figure 5 shows the longitudinal microstructures of directionally solidified hypereutectic Al-12 wt.%Ni alloys at various growth rates under a 0.1 T magnetic field. The measurement results show that the area percentages of the primary Al_3_Ni phase respectively are 30%, 28%, 26% and 22% for Fig. 5(a–d) when the growth length is 5 mm. When the growth length increases to 15 mm, the area percentages of the primary Al_3_Ni phase respectively are 25%, 23%, 18% and 15%. The area percentages of the primary Al_3_Ni phase respectively are 1%, 5%, 10% and 13% when the growth length increase to 25 mm. It can be seen that the enrichment degree of the primary phase decreases with the increase of the growth rate. Thus, the graded composition of the primary Al_3_Ni phases is weakened by the growth rate. Figure 6 shows the longitudinal microstructures of directionally solidified hypereutectic Al-96 wt.%Zn alloys at a 5 μm/s growth rate under a 0.5 T magnetic field at the temperature gradient of 60 K/cm and 120 K/cm. The measurement results show that the area percentages of the primary Zn phase respectively are 56% and 60% for Fig. 6a,b when the growth length is 5 mm. When the growth length increases to 15 mm, the area percentages of the primary Zn phase respectively are 43% and 49%. The area percentages of the primary Zn phase respectively are 22% and 10% when the growth length increase to 25 mm. The enrichment degree of the primary phase increases with the increase of the temperature gradient. Therefore, the graded composition of the primary Zn phases is enhanced by the temperature gradient.

**Figure 7:**
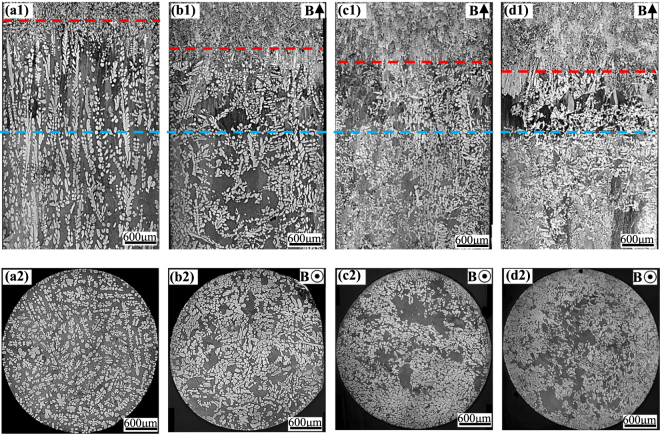
The longitudinal and corresponding transverse microstructures near the solid/liquid interface in directionally solidified Al-96 wt.%Zn alloy at a 7 μm/s growth rate under various magnetic fields. (**a**) 0 T; (**b**) 1 T; (**c**) 3 T; (**d**) 5 T. Blue and red dotted lines marking the bottom and the top of the mushy zone, respectively.

**Figure 8:**
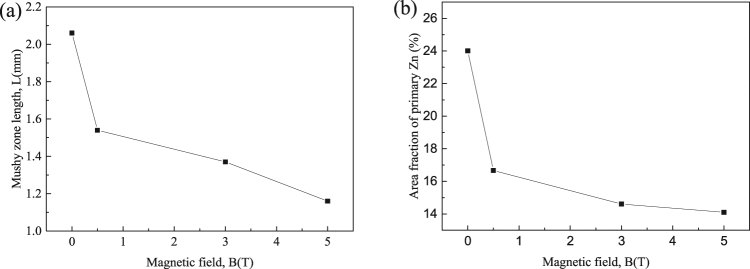
The effect of the magnetic field on the mushy zone length and the area of primary phase in a directionally solidified Al–96 wt.%Zn alloy at a 7 μm/s growth rate. (**a**) The mushy zone length as a function of the magnetic field; (**b**) the area fraction of primary phase as a function of the magnetic field.

In order to study the origin of the graded composition profile in FGMs during directional solidification under the magnetic field, the microstructures near the solid/liquid interface in hypereutectic Al-96 wt.%Zn alloys at a 7 μm/s growth rate under various magnetic fields were examined. Figure 7 shows the longitudinal microstructures near the liquid/solid interface and corresponding transverse microstructures of 5 mm under the liquid/solid interface. The measure of the mushy length and the area percentage of the primary phase of the mushy zone in the longitudinal section in Fig. 7 are shown in Fig. 8. It can be seen that the mushy length and the area percentage of the primary phase decrease with increase of the magnetic field.

Moreover, one can notice that the dendritic morphology is typical columnar microstructure in the absence of the magnetic field, as shown in Fig. 7. However, the applied magnetic field causes the regular dendritic microstructure to be destroyed and the columnar-to-equiaxed transition (CET). It can be seen that, in the mushy zone, the equiaxed crystal amounts with the increase of magnetic field. When the magnetic field of 5 T is applied, the microstructure almost transforms into equiaxed crystals.

## Discussions

### TE magnetic convection in the liquid and TE magnetic force on the solid under an axial static magnetic field

Owing to the Seebeck effect, the difference in TE powers of the solid phase and liquid phase near the solid/liquid interface induces a TE current during directional solidification. Thus, in the system, the applied magnetic field will produce a TE magnetic force which causes a TE magnetic convection in the liquid. In order to investigate the distribution and magnitude of the TE magnetic convection in the liquid and the TE magnetic force acting on the solid, Numerical simulation was performed by using the finite element commercial code COMSOL Multiphysics. The temperature field, TE current density, fluid flow field and magnetic field intensity are coupled in the simulation. The basic equations of the numerical simulation are

1 $$\overrightarrow{J}=\sigma (\overrightarrow{E}+\overrightarrow{u}\times \overrightarrow{B}-S\overrightarrow{\nabla }T)$$

2 $$\overrightarrow{\nabla }\cdot \overrightarrow{J}=0$$

3 $$\overrightarrow{\nabla }\cdot \overrightarrow{u}=0$$

where $\overrightarrow{j}$, *σ*, *E*, *u*, *B*, *S* and $\overrightarrow{{\nabla }}$*T* respectively denote the electric current density, electric conductivity, electric field, fluid velocity, magnetic field intensity, TE power and temperature gradient.

4 $$\overrightarrow{\nabla }T={G}_{\infty }{i}_{Z}$$

where *G*_∞_ is a constant of 90 K/cm, and *i*_z_ is a unit vector along the z-axis.

5 $$\overrightarrow{\nabla }{T}_{i}=0$$

where i = l or s in the liquid or in the solid, respectively. More details about the equations and corresponding boundary conditions can be seen in ref.^24^. The physical properties used in the computations are listed in Table 1.

**Table 1:** Physical properties and parameters used in numerical simulation.

Properties	Unite	Al-Cu		Al-Zn	
		Solid	Liquid	Solid	Liquid
Density (ρ)	kg/m^3^	2.7 × 10^3^	2.4 × 10^3^	7.14 × 10^3^	6.3 × 10^3^
Dynamic viscosity (μ)	Pa s	—	2.9 × 10^−3^	—	3.1 × 10^−3^
Electrical conductivity (σ)	S/m	7.9 × 10^7^	4.0 × 10^6^	1.7 × 10^7^	3.72 × 10^7^
Thermoelectric (S)	V/K	−1.5 × 10^−6^	−2.25 × 10^−6^	1.83 × 10^−7^	1.0 × 10^−7^

**Figure 9:**
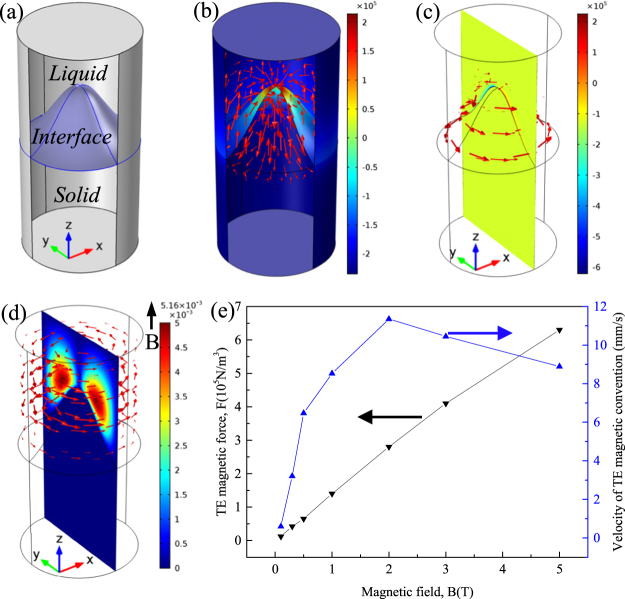
Numerical simulation of the TE magnetic effects under a 1 T magnetic field for the Al-Cu alloy. (**a**) 3-D geometry; (**b**) red arrows show the 3-D distribution of computed TE current around the solid/liquid interface (the unit in caption is A/m^2^); (**c**) TE force near the solid/liquid interface (the unit in caption is N/m^3^); (**d**) TE magnetic convection near the solid/liquid interface (the unit in caption is m/s); (**e**) the maximum value of the computed TE magnetic force and TE magnetic convection near the solid/liquid interface as a function of the magnetic field.

**Figure 10:**
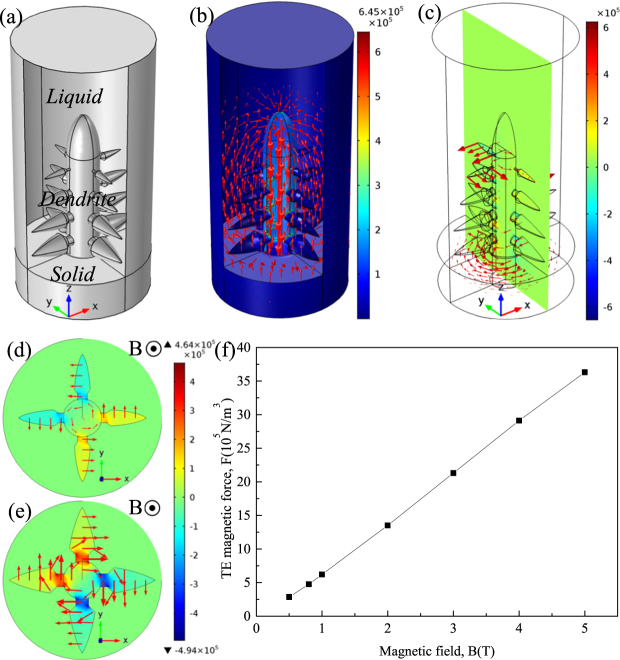
Numerical simulation of the TE magnetic effects under a 1 T magnetic field for the Al-Zn alloy. (**a**) 3-D geometry; (**b**) red arrows show the 3-D distribution of computed TE current in the dendrite (the unit in caption is A/m^2^); (**c**) TE force acting on the dendrite (the unit in caption is N/m^3^); (**d**,**e**) the distribution of the TE magnetic force on the x-y plane at the top and bottom of the columnar dendrite (the unit in caption is N/m^3^); (**f**) the maximum value of the computed TE magnetic force acting on the columnar dendrite as a function of the magnetic field.

In order to study the influence of TE magnetic convection on the solidification structure, the numerical simulation at a sample scale was carried out. Figure 9 shows the 3-D geometry and typical distribution of the TE current, TE magnetic force and TE magnetic convection at a sample scale under a 1 T magnetic field for Al-Cu alloy. The TE current forms the circuits around the interface (Fig. 9b). Figure 9c,d show the computed TE magnetic force acting on the liquid and the corresponding TE magnetic convection. Furthermore, one can notice that the TE magnetic convection first reaches the maximum under a 2 T magnetic field, and then decreases with the increase of the magnetic field (Fig. 9e). In order to study the effect of TE magnetic force on the solidification structure, the numerical simulation at a single dendritic scale was carried out. Figure 10 shows the 3-D geometry and typical distribution of the TE current and TE magnetic force at a single dendritic scale under a 1 T magnetic field for Al-Zn alloy. The TE current forms the circuits around the dendrite (Fig. 10b). Figure 10c,d and f show the distribution of the computed TE magnetic force. It can be seen that the TE magnetic forces respectively orientate anticlockwise and clockwise at the top and bottom of the columnar dendrite. Thus, a torque forms on the columnar dendrite. Figure 10f shows that the TE magnetic force on the solid is enhanced by the magnetic field.

The TE magnetic convection under an axial magnetic field during directional solidification is similar to the rotating convection driven by a low-frequency low-induction rotating magnetic field^25,26^. When the Taylor number has a magnitude of 10^5^ according to the aspect ratio of the radius/height, the convection is laminar. However, for the TE magnetic convection during directional solidification, the Taylor number is estimated to be 1.35 × 10^7^ under a 2 T magnetic field. The Taylor number can be derived by the non-dimensional parameters of Hartmann number and Reynolds number, given by *Ha* = *BR* (*σ*/(*ρυ*))^1/2^ and *Re*_*w*_ = *ρUR*/*μ*, respectively.

6 $$Ta=H{a}^{2}R{e}_{w}=\frac{\sigma {B}^{2}u{R}^{3}}{\mu \upsilon }$$

where *R*, *ρ*, *μ and υ* denote the inner radius of crucible, density of the melt, dynamic viscosity and kinematic viscosity of the melt, respectively. Ta increases with the TE magnetic convection and magnetic field. When Ta is higher than 10^5^, Taylor–Görtler vortices will form. The vortex is a circulation in the longitudinal section of the sample near the mushy zone during directional solidification under the magnetic field, which is very effective for the upward and downward migration of the species.

### Formation mechanism of compositional gradient under an axial static magnetic field

According to the above experiment and simulation results, two mechanisms are assumed to be responsible for the formation of compositional gradient:

1. The heavier species (Zn, Ni and Cu) migrate downward under the gravitation force during directional solidification, which is beneficial to the enrichment of heavier species at the bottom of the samples. Thus, the compositional gradient will form and the graded composition of the primary phases occurs.
2. The circulation as mentioned above transfers the higher temperature bulk melt downward under the magnetic field during directional solidification. Due to the existence of axial temperature gradient, it is beneficial to the growth of the primary phases which have been precipitated at the bottom of the samples. Therefore, the graded composition of the primary phases will form.

**Figure 11:**
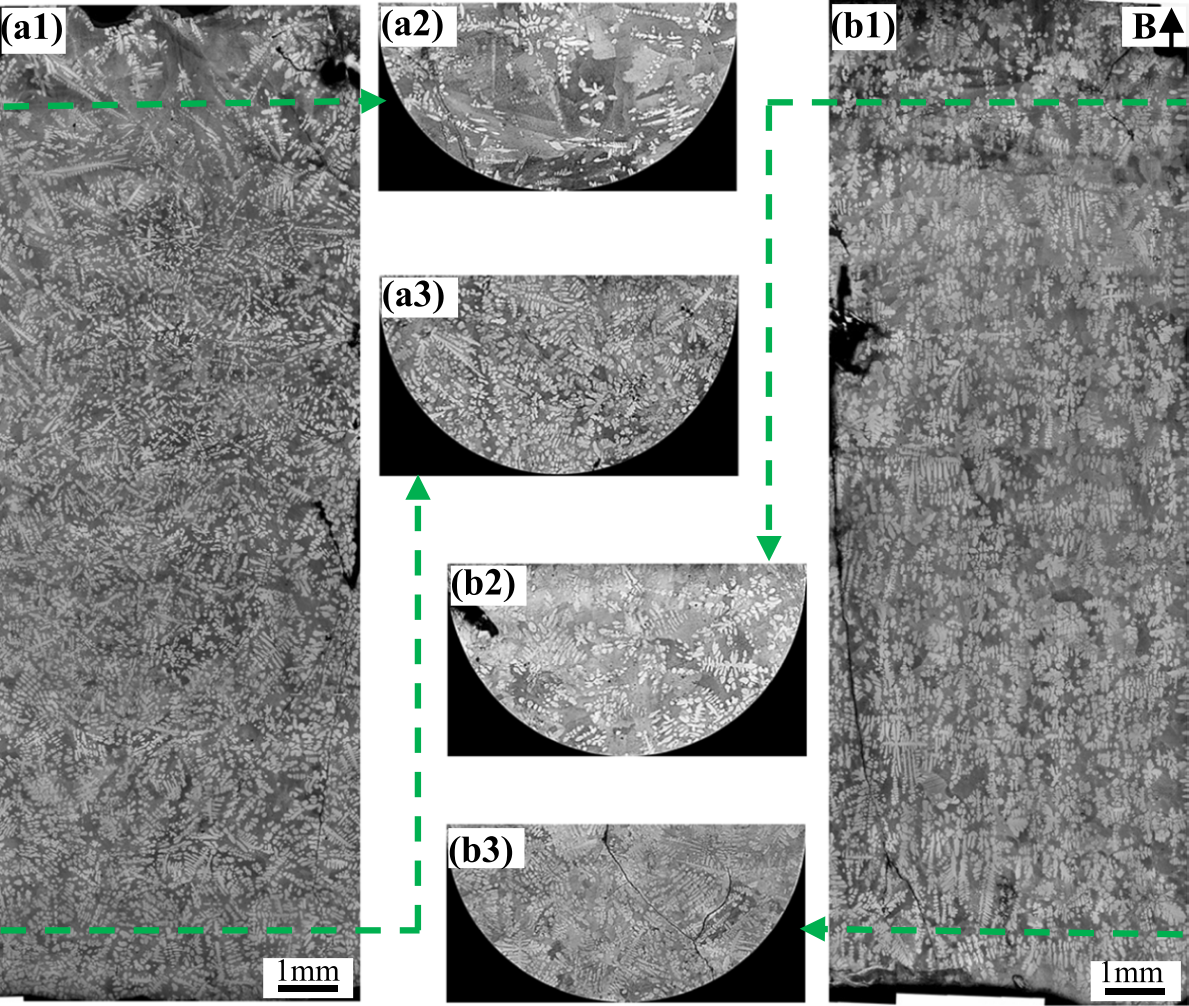
The effect of a 5 T static axial magnetic field on the microstructures in solidified Al-96 wt.%Zn alloys at a cooling rate of 3 K/min. (**a**) The longitudinal and the corresponding transverse microstructures without magnetic field at the top and bottom of the sample; (**b**) the longitudinal and corresponding transverse microstructures with magnetic field at the top and bottom of the sample.

To figure out the formation mechanism of the graded composition of the primary phases during directional solidification under the magnetic field, a volume solidification experiment was carried out. The Al-96 wt.%Zn alloy sample was first heated to 873 K for 1 h in the constant temperature zone of the furnace, and then cooled at a cooling rate of 3 K/min without and with a 5 T magnetic field. The experimental results are shown in Fig. 11. Figure 11a shows the distribution of the primary phase without the magnetic field. It can be seen that the graded composition of the primary phases occurs. The applied 5 T magnetic field suppresses the graded composition of the primary phases. The above results suggest that the graded composition of the primary phases results from the gravity force. In the absence of the magnetic field, the downward migration of heavier Zn species under the gravity force and the graded composition of the primary phases will form. The application of the magnetic field prevents the migration of the heavier Zn species. Therefore, the downward migration of the heavier species under the gravity force is one of the reasons leading to the graded composition of the primary phases during vertical directional solidification.

**Figure 12:**
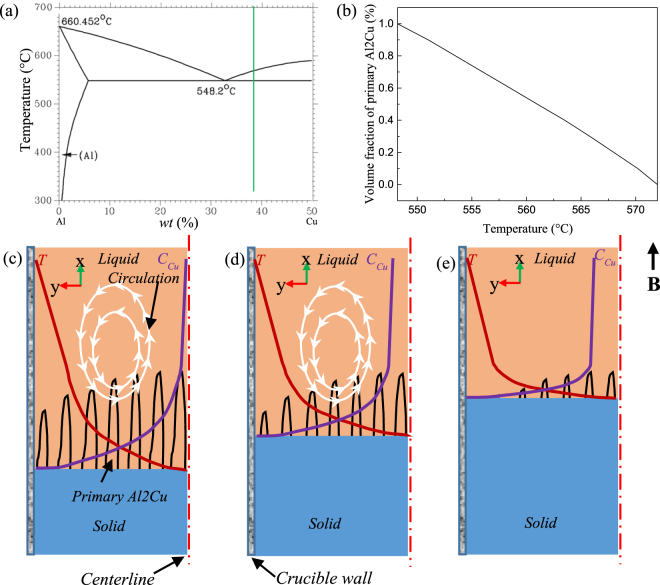
Schematic illustration of the compositional gradient mechanism of Al–40 wt.%Cu alloy during directional solidification under an axial static magnetic field. (**a**) The equilibrium phase diagram of Al-Cu alloy; (**b**) the volume fractions of the precipitated primary Al_2_Cu phase as a function of the temperature; (**c**) the distribution of the temperature and Cu content near the solid/liquid interface and the circulation due to the applied magnetic field; (**d**) the decrease of the Cu content in the remaining melt and the length of the mushy zone induced by circulation; (**e**) the formation of the Al-Cu eutectic and disappearing of the mushy zone and the circulation with the decrease the Cu content in the remaining melt.

The other results above suggest that the graded composition of the primary phases under the magnetic field during directional solidification may be attributed to the TE magnetic convection. Figure 12 shows a schematic illustration of the mechanism of the compositional gradient in the directionally solidified Al-40 wt.%Cu alloy under an axial static magnetic field. Figure 12a,b respectively show the equilibrium phase diagram of the Al-40 wt.%Cu alloy and the volume fractions of the precipitated primary Al_2_Cu phase at the different temperatures. It can be seen that the volume fractions of the precipitated primary Al_2_Cu phase decrease with the increase of the temperature. Therefore, the volume fractions of the primary Al_2_Cu phase along the longitudinal section in the melt are different. In the present work, a higher temperature gradient exists along the longitudinal direction during directional solidification. This is beneficial for the precipitation of the primary Al_2_Cu phase at the bottom of the sample. When the magnetic field is applied, the circulation mentioned above will appear. The rich Cu melt in the upper local part is taken to the mushy zone, as shown in Fig. 12c, which is useful to the formation and growth of the primary Al_2_Cu phase. Moreover, the viscosity of the liquid metal near the mushy zone increases with the decrease of the temperature and the increase of the primary Al_2_Cu phase amount. Both reasons keep a rapid growth of the primary Al_2_Cu phase. As the directional solidification progresses under the magnetic field, the local melt in the upper part of the mushy zone can be carried downward to the mushy zone to fulfil the growth of the primary Al_2_Cu phase. The Cu content of the local melt in the upper part of the mushy zone will be far less than the equilibrium concentration of the Al-40 wt.%Cu alloy. Thus, the Cu concentration gradient will form. Meanwhile, the rich Cu melt which is far away from mushy zone migrates downward to the local melt in the upper part of the mushy zone under the concentration gradient and the gravity force. The downward migration of Cu in the melt can ensure the continuous growth of the primary Al_2_Cu phase. Moreover, when the Cu content in the remaining melt decreases, the growth of the primary Al_2_Cu phase is inhibited and the length of the mushy zone is decreased, as shown in Fig. 12d. The remaining melt in the directionally solidified Al-40 wt.%Cu gradually becomes poor Cu melt, which is extended to the eutectic content. The primary Al_2_Cu phase tends to disappear, which causes the TE magnetic convection and the corresponding circulation to vanish, as shown in Fig. 12e. Finally, the Al-40 wt.%Cu alloy will be solidified with the eutectic composition and the graded composition of the primary phases will form.

The above analyses confirm that the graded composition of the primary phases during directional solidification should be attributed to the combined action of heavier species migration under the gravity force and TE magnetic convection under the magnetic field. Moreover, the circulation caused by the TE magnetic convection promotes the heavier species migration, which enhances the graded composition of the primary phases.

Furthermore, one can notice that the graded composition of the primary phases is enhanced by the magnetic field for the Al-96 wt.%Zn alloy during directional solidification. However, the graded composition of the primary phases is enhanced first and then weakened by the magnetic field for the Al-12 wt.%Ni and Al-4  wt.%Cu alloys. This phenomenon is related to two types of Lorentz forces. One is generated by the internal electric current, due to TE effects, and promotes convection, while the other is generated by the induced electric current, due to electromagnetic (EM) induction, and damps convection. The latter will play a major role with the increase of magnetic field. Thus, when the magnetic field increases to a certain value, the TE magnetic convection decreases. The magnetic field *B*_max_, which corresponds to the maximum of the TE magnetic convection, can be written as^27^

7 $${B}_{max}={(\frac{\rho S\nabla T}{\lambda \sigma })}^{1/3}$$

where *λ* donates a typical length scale. As shown in Fig. 1, *B*_*max*_ ≥ 5 T and the graded composition of the primary phases is enhanced by the magnetic field for the Al-96 wt.%Zn alloy during directional solidification. The results in Figs 7 and 8 show that the mushy length and the area fraction of the primary phase decrease with the increase of the magnetic field. This indicates that the downward migration of the heavier species and the circulation are accelerated, which increases the graded composition of the primary phases. However, for the Al-12 wt.%Ni and Al-40 wt.%Cu alloys, *B*_*max*_ = 1 the graded composition of the primary phases is weakened by the magnetic field when the magnetic field is more than 1 T, as shown in Figs 2 and 3. The simulation results in Fig. 9 show that the maximum of the TE magnetic convection can be reached for the Al-Cu alloy when the 2 T magnetic field is applied. Although the growth rate is ignored in numerical simulation, it can be seen that the results are in rough agreement with the experimental results at a lower growth rate for the directionally solidified Al-40 wt.%Cu alloy.

### Formation mechanism of equiaxed FGMs under an axial static magnetic field

The experimental results in Fig. 7 show that the applied magnetic field can be used to fabricate the equiaxed FGMs in the directionally solidified Al-96 wt.%Zn alloys. It is well known that a TE magnetic force can not only act on the liquid to generate convection but also act on the solid to generate torque on the dendrite during directional solidification under the magnetic field. The previous studies have shown that CET under the static magnetic field is not induced by the TE magnetic convection but the TE magnetic force^16,28^. The TE magnetic force acting on the solid can be written as^19^.

8 $$F=2{\sigma }_{S}B\nabla T[\frac{{\sigma }_{l}}{2{\sigma }_{l}+{\sigma }_{S}}]({S}_{s}-{S}_{l})$$

where *σ*_*s*_, *σ*_*l*_, *S*_*s*_ and *S*_*l*_ respectively denote the electrical conductivity in solid, electrical conductivity in liquid, thermoelectric power in solid and thermoelectric power in liquid. Figure 10 shows the simulation results of the amplitude and distribution of the TE magnetic force acting on a single columnar dendrite. One can notice that the TE magnetic force is mainly concentrated at the dendrite tip and the root of the primary dendrite arm and the secondary dendritic arm. When the magnetic field is applied, the TE magnetic force is up to the order of 3.6 × 10^6^ N/m^3^ under a 5 T magnetic field. This force is strong enough to break dendrite and cell^28^. Moreover, TE magnetic force orientates respectively anticlockwise and clockwise at the top and bottom of the columnar dendrite, as shown in Fig. 10d,e. A strong torque will be produced on the primary dendrite. Due to the torque on the primary dendrite and the necking at the root of the secondary dendritic arm, the dendritic is supposed to break and the CET will occur. Thus, the equiaxed FGMs are successfully produced.

## Conclusions

The present work describes an *in situ* technique to fabricate Al alloy FGMs (i.e., Al-Zn, Al-Ni and Al-Cu alloys) using directional solidification process under an axial static magnetic field. It can be found that the graded composition of the primary phases (i.e., Zn, Al_3_Ni and Al_2_Cu) is obvious along the longitudinal section of the sample. The graded composition of the primary phases is enhanced first and then weakened when the applied magnetic field is enough strong. This phenomenon is related to two types of Lorentz forces. One promotes convection while the other damps convection. The graded composition of the primary phases can also be increased by the decrease of the growth rate and increase of the temperature gradient under the magnetic field. A proposed model and simulations are carried out to explain the origin of the graded composition of the primary phases in FGMs during directional solidification under an axial static magnetic field. It should be attributed to the combined actions of heavier species migration under the gravity force and TE magnetic convection under the magnetic field. Furthermore, the magnetic field can induce columnar FGMs to change into equiaxed FGMs. Therefore, the application of the axial static magnetic field during directional solidification can be used as an effective method to fabricate FGMs.
